# Development and Validation of a New Clinical Prediction Model of Catheter-Related Thrombosis Based on Vascular Ultrasound Diagnosis in Cancer Patients

**DOI:** 10.3389/fcvm.2020.571227

**Published:** 2020-10-26

**Authors:** Binliang Liu, Junying Xie, Xiaoying Sun, Yanfeng Wang, Zhong Yuan, Xiyu Liu, Zhou Huang, Jiani Wang, Hongnan Mo, Zongbi Yi, Xiuwen Guan, Lixi Li, Wenna Wang, Hong Li, Fei Ma, Yixin Zeng

**Affiliations:** ^1^Department of Medical Oncology, National Cancer Center/National Clinical Research Center for Cancer/Cancer Hospital, Chinese Academy of Medical Sciences and Peking Union Medical College, Beijing, China; ^2^Department of Management, Cancer Hospital of Huanxing, Beijing, China; ^3^Department of Medical Oncology, Cancer Hospital of Huanxing, Beijing, China; ^4^Department of Comprehensive Oncology, National Cancer Center/National Clinical Research Center for Cancer/Cancer Hospital, Chinese Academy of Medical Sciences and Peking Union Medical College, Beijing, China; ^5^Vascular Access Center, Hunan Cancer Hospital/The Affiliated Cancer Hospital of Xiangya School of Medicine, Central South University, Changsha, China; ^6^Department of Lymphoma and Hematology, Hunan Cancer Hospital/The Affiliated Cancer Hospital of Xiangya School of Medicine, Central South University, Changsha, China; ^7^Key Laboratory of Carcinogenesis and Translational Research (Ministry of Education/Beijing), Department of Radiation Oncology, Peking University Cancer Hospital and Institute, Beijing, China; ^8^Department of Cardiology, Beijing Anzhen Hospital, Capital Medical University, Beijing, China; ^9^Chinese Academy of Medical Sciences and Peking Union Medical College, Beijing, China; ^10^State Key Laboratory of Oncology in South China, Sun Yat-sen University Cancer Center, Collaborative Innovation Center for Cancer Medicine, Guangzhou, China

**Keywords:** catheters, thrombosis, nomogram, risk factor, cancer

## Abstract

**Background:** Central venous catheters are convenient for drug delivery and improved comfort for cancer patients, but they also cause serious complications. The most common complication is catheter-related thrombosis (CRT).

**Objectives:** This study aimed to evaluate the incidence and risk factors for CRT in cancer patients and develop an effective prediction model for CRT in cancer patients.

**Methods:** The development of our prediction model was based on a retrospective cohort (*n* = 3,131) from the National Cancer Center. Our prediction model was confirmed in a prospective cohort from the National Cancer Center (*n* = 685) and a retrospective cohort from the Hunan Cancer Hospital (*n* = 61). The predictive accuracy and discriminative ability were determined by receiver operating characteristic (ROC) curves and calibration plots.

**Results:** Multivariate analysis demonstrated that sex, cancer type, catheter type, position of the catheter tip, chemotherapy status, and antiplatelet/anticoagulation status at baseline were independent risk factors for CRT. The area under the ROC curve of our prediction model was 0.741 (CI: 0.715–0.766) in the primary cohort and 0.754 (CI: 0.704–0.803) and 0.658 (CI: 0.470–0.845) in validation cohorts 1 and 2, respectively. The model also showed good calibration and clinical impact in the primary and validation cohorts.

**Conclusions:** Our model is a novel prediction tool for CRT risk that accurately assigns cancer patients into high- and low-risk groups. Our model will be valuable for clinicians when making decisions regarding thromboprophylaxis.

## Introduction

With the rapid growth of medical research over the past decades, more advanced cancer treatments are available for cancer patients to improve their survival. With advances in cancer treatments, more complications, especially cardiovascular complications, have been widely seen in practice (1, 2). To provide better cardiovascular care for cancer patients, a new multidisciplinary field of cardio-oncology was established and has received appreciation and recognition worldwide (3).

Central venous catheters were considered a medical advance that brought convenience for drug delivery and improved comfort for patients, but it also introduced catheter-related complications (4, 5). Catheter-related thrombosis (CRT) is one of the major catheter-related complications affecting many cancer patients (5). The reported incidences of CRT range from 2.4 to 61.5% in cancer patients (4–10). Unlike cancer-associated venous thromboembolism, CRT is recognized as a unique entity because the incidence of CRT in cancer patients is correlated with cancer- and catheter-related risk factors for thrombus formation (11). Moreover, catheter-related risk factors play critical roles that cannot be neglected (6). The central venous catheter, one device, contributes to the three factors of venous thrombus formation described in Virchow's triad (12): the placement of a venous catheter can cause local vessel damage; the presence of a venous catheter changes the dynamics of blood flow (13); and protein and blood cell adhesion on the surface of the catheter increased hypercoagulability. Due to the high incidence of CRT in cancer patients, several clinical trials were initiated to test the efficacy of routine thromboprophylaxis for CRT prevention. However, the results were not conclusive or solid enough to support routine thromboprophylaxis for cancer patients with catheters (11). Therefore, the decision to apply preventive treatment with anticoagulants should be discussed case by case.

The previously published results of the AVERT trial and the CASSINI trial showed benefits of thromboprophylaxis in cancer patients with a high risk of venous thromboembolism (14, 15). Venous thromboembolism (VTE) risk stratification in these two trials was based on the well-validated risk prediction tool, the Khorana risk score (16). While routine thromboprophylaxis for VTE prevention in the general patient population is controversial (17), positive results from these trials have proven the applicability of risk stratification to identify patients who are most likely to benefit from thromboprophylaxis.

CRT is defined as VTE associated with the use of a central venous catheter (18). The same strategy of risk stratification could be applied to CRT prevention in cancer patients. However, first, a well-designed and validated risk prediction tool is needed. We conducted a large-scale observational cohort study of cancer patients with catheters to develop a CRT risk prediction model. In addition to the main cancer-related factors considered in the Khorana risk score, catheter-related factors were also largely investigated when developing our model. Both symptomatic and asymptomatic CRT were considered in our analysis because they have similar clinical impacts on the prognosis of cancer patients (19). Validation of this model was performed in two independent external cohorts.

## Materials and Methods

### Study Subjects and Study Design

The primary cohort consisted of cancer patients treated at the National Cancer Center/National Clinical Research Center for Cancer/Cancer Hospital, Chinese Academy of Medical Sciences and Peking Union Medical College between January 1, 2015 and December 31, 2018. Patients in validation cohort 1 were recruited from the center mentioned above between January 1, 2019 and August 31, 2019, prospectively and independently. Patients in validation cohort 2 were recruited from Hunan Cancer Hospital, and their data were analyzed retrospectively.

The inclusion criteria of patients in our study were as follows: (1) adult and ambulatory patients who were pathologically diagnosed with malignant tumors and underwent successful catheterization, (2) patients who voluntarily participated and voluntarily reported their data in this study, and (3) at least one vascular ultrasound examination was performed during catheter placement. The exclusion criteria were as follows: (1) incomplete basic patient information, (2) the catheter had not been removed at the beginning of patient screening for the primary cohort and validation cohort 2 or at the end of follow-up for validation cohort 1, and (3) unknown location of the primary tumor.

According to the maximum duration of use of centrally inserted central catheters (CICCs) or peripherally inserted central catheters (PICCs), our primary endpoint was objectively confirmed CRT in patients with CICCs during the 3-months use period and in patients with PICCs during the 12-months use period. Patients in validation cohort 2 were under continuous follow-up, and all variables were recorded until extubation was performed upon the doctor's request or when thrombosis occurred (whichever occurred first). The diagnosis of CRT was made by vascular ultrasound.

Thirty-six variables were recorded, and these variables included (but were not limited to) the following: general information (age, sex, body mass index, and smoking and drinking habits); past or concomitant diseases [hypertension, diabetes mellitus, coronary heart disease, cerebral infarction, deep venous thrombosis (DVT), and arrhythmia]; cancer status (tumor type, stage, Karnofsky performance score); baseline treatment information; catheter-related information (purpose of catheterization, catheterization history, catheter type, insertion side, position of the catheter tip, and secondary adjustment of the catheter); and baseline examination (routine blood test, D-dimer test). All 36 variables are listed in Table 1 and Supplementary Table 1 (baseline examination).

**Table 1:** Baseline patient characteristics.

**No**.	**Characteristic**	**Primary cohort (*n* = 3,131)**	**%**	**Validation cohort 1 (*n* = 685)**	**%**	**Validation cohort 2 (*n* = 61)**	**%**
**Demographic and patient-related characteristics**							
1	Age						
	<60	2,118	67.65	456	66.57	51	83.61
	≥60	1,013	32.35	229	33.43	10	16.39*
2	Sex						
	Male	997	31.84	266	38.83*	29	47.54*
	Female	2,134	68.16	419	61.17*	32	52.46*
3	KPS						
	>80 points	2,507	80.07	604	88.18*	60	98.36*
	≤ 80 points	624	19.93	81	11.82*	1	1.64*
	Comorbidity						
4	Hypertension	752	24.02	182	26.57	10	16.39
5	Diabetes mellitus	342	10.92	81	11.82	5	8.20
6	Coronary heart disease	114	3.64	33	4.82	1	1.64
7	Cerebral infarction	61	1.95	25	3.65*	0	0.00
8	Deep venous thrombosis	15	0.48	3	0.44	0	0.00
9	Arrhythmia	84	2.68	28	4.09	0	0.00
10	Smoking history	751	23.99	155	22.63	20	32.79
11	Drinking history	697	22.26	141	20.58	16	26.23
12	BMI						
	≥25	1,266	40.43	421	61.31*	40	65.57*
	<25	1,865	59.57	264	38.54*	21	34.43*
**Cancer-related characteristics**							
13	Type of cancer						
	Breast cancer	1,433	45.77	242	35.33*	16	26.23*
	Thoracic cancers	663	21.18	164	23.94	1	1.64*
	Gastrointestinal cancers	530	16.93	154	22.48*	6	9.84
	Urogenital cancers	372	11.88	98	14.31	8	13.11
	Hematological cancers	88	2.81	16	2.34	7	11.48*
	Other cancers^#^	45	1.44	11	1.61	23	37.70*
14	Stage of cancer						
	Localized tumor (stages I–III)	1,490	47.59	397	57.96*	0	0.00*
	Advanced tumor (stage IV)	1,641	52.41	288	42.04*	61	100.00*
**Treatment-related characteristics**							
15	Chemotherapy (conventional or targeted)	2,863	91.44	610	89.05	57	93.44
16	Radiotherapy	408	13.03	149	21.75*	4	6.56
17	Parenteral nutrition	120	3.83	8	1.17*	0	0.00
18	Anti-infective therapy	359	11.47	21	3.07*	1	1.64*
19	Symptomatic support (excluding parenteral nutrition) and pretreatment before surgery or radiotherapy	57	1.82	26	3.80*	9	14.75*
20	Anticoagulation treatment	15	0.48	3	0.44	0	0.00
21	Antiplatelet treatment	39	1.25	14	2.04	1	1.64
**Catheter-related characteristics**							
22	Catheter days (days)						
	0–44 days	976	31.17	320	46.72*	7	11.48*
	45–89 days	861	27.50	180	26.28*	23	37.70*
	90–365 days	1,294	41.33	185	27.01*	31	50.82*
23	Catheter days, total	221,074		38,144		5,991	
24	Catheter days, median (range)	68.0 (0–345)		48.0 (0–181)		90.0 (6–259)	
25	Catheter days, mean (*SD*)	70.6 ± 44.7		55.7 ± 34.3*		98.2 ± 50.1*	
26	Type of venous catheter						
	CICC	1,621	51.77	468	68.32*	2	3.28*
	PICC	1,510	48.23	217	31.68*	59	96.72*
27	Insertion side of catheter						
	Left	921	29.42	172	25.11	59	96.72
	Right	2,210	70.58	513	74.89	2	3.28
28	Position of the catheter tip						
	Proper position (T6–8)	2,849	90.99	654	95.47*	55	90.16
	Improper position						
	Above T6–8	234	7.47	18	2.63*	6	9.84
	Under T6–8	29	0.93	0	0.00	0	0.00
	Not in the superior vena cava	19	0.61	13	1.90*	0	0.00
29	Secondary adjustment of catheter position	73	2.33	10	1.46	0	0.00
30	Previous history of catheterization	555	17.73	163	23.80*	1	1.64*

*NA, not available*.

# *Head and neck cancer, melanoma, sarcoma, neuroendocrine tumor*.

* *Differences between the primary and validation cohorts, p < 0.05*.

All patients received routine catheter care provided by a professional team once or twice per week. During follow-up visits, patients in validation cohort 2 were asked about their general situation and whether they had experienced any adverse events or complications since the last visit. Information about complications was continuously recorded.

This study did not interfere with any doctor's decision-making and did not change or delay any treatments.

### Catheterization Method

Modified Seldinger technique with ultrasound guidance was used for CICC and PICC placement. Detailed catheterization methods are shown in the Supplementary Textual Material. All CICCs were non-tunneled subclavian catheters.

After catheterization, a chest X-ray examination (including the upper limb and neck on the ipsilateral side of the catheter) was performed to confirm the catheter's direction and the position of the catheter tip. All X-ray films were evaluated by a physician specializing in venous catheterization and at least one radiologist responsible for chest X-ray reports. The tip of the catheter was typically located at the lower third of the superior vena cava, the cavoatrial junction, or the upper third of the right atrium. If the vertebrae were used as a reference, being at thoracic vertebra segment 6–8 (T6–T8) was considered the proper position; otherwise, it was deemed improper.

### Doppler Ultrasound

Ultrasound with Doppler and color imaging (GE LOGIQ^TM^ E9) was performed at extubation and if any clinical symptoms suggesting CRT were noted.

Every ultrasound report was evaluated by the same radiologist team at the National Cancer Center. CRT was diagnosed after finding a thrombus with partial or total occlusion of the vessel.

## Statistical Analysis

### General Characteristics and Incidence Reports

The incidence of CRT was calculated as the total number of catheter-related thromboses divided by the total number of catheters placed (%) or divided by 1,000 catheter days (/1,000 days of use).

Pearson's *x*^2^ test was used to compare categorical variables, and an independent samples *t*-test was used to compare continuous variables. All statistical tests were two-sided, and *p* < 0.05 were considered statistically significant. Pearson's *x*^2^ test and the independent samples *t*-test were performed with SPSS software (Version 23, SPSS Inc., IBM, NY, USA).

### Development of the Prediction Model

Univariable and multivariable analyses were performed to identify the significant independent risk factors for CRT. Variables with *p* < 0.25 in the univariate analysis were included in the multivariate analysis (20). The results are presented as the adjusted odds ratios (ORs) with 95% confidence intervals (CIs). The prediction model was developed based on the results of the multivariate analysis by binary logistic regression and further optimized by stepwise forward and backward selection. A relatively optimal model was ultimately achieved. A nomogram was formulated to illustrate our prediction model by using the rms package (version 5.1-4) in R software (http://www.r-project.org/, The R Foundation for Statistical Computing v3.6.0). The univariable, multivariable, and binary logistic regression analyses were performed with SPSS software (Version 23, SPSS Inc., IBM, NY, USA).

### Assessment of the Prediction Model

The performance of the model was assessed in terms of discrimination, calibration, and clinical impact by using the packages rms (version 5.1-4), pROC (version 1.15.3), and rmda (version 1.6) in R software (http://www.r-project.org/, The R Foundation for Statistical Computing v3.6.0).

Discrimination was measured by the area under the receiver operating characteristic (ROC) curve. Calibration was measured by calibration plots, the coefficient of determination (*R*^2^), and the Hosmer–Lemeshow (H-L) test (21). The cutoff value of our model between the high- and low-risk groups was derived from the maximum Youden index. Furthermore, clinical impact was assessed by decision curve analysis (22).

## Results

### Patient Characteristics

From January 1, 2015 to December 31, 2018, we enrolled 3,860 cancer patients who received a catheter and underwent a vascular ultrasound examination. After screening, 3,131 patients were enrolled in the primary cohort. The total number of catheter days was 221,074, and the median number of catheter days per patient was 68.0 (range, 0–345). For validation cohort 1, 2,909 patients were recorded, and 685 patients were ultimately enrolled. Validation cohort 2 consisted of 61 patients. The flow chart of patient enrollment is shown in Supplementary Figure 1.

Among 3,131 patients in the primary cohort, 2,134 (68.16%) were women, and the mean patient age (standard deviation, *SD*) was 53.7 (±11.1) years. Among 685 patients in validation cohort 1, 419 (61.17%) were women, and the mean patient age (SD) was 54.2 (±11.7) years. Among 61 patients in validation cohort 2, 32 were women, and the mean patient age (SD) was 59.2 (±11.0) years. The characteristics of patients, such as chemotherapy and antiplatelet or anticoagulation status at baseline, were balanced across the primary and validation cohorts. However, as shown in Table 1, the percentage of female patients was significantly higher in the primary cohort than in the validation cohorts (68.16 vs. 61.17% in validation cohort 1 and 52.46% in validation cohort 2). The proportion of breast cancers was also higher in the primary cohort (45.77%) than in the validation cohorts (35.33% in validation cohort 1 and 26.23% in validation cohort 2). The three populations differed notably concerning the type of venous catheter used and the position of the catheter tip. The D-dimer concentration was not requested when collecting data on the patients in the validation cohorts because it was not a significant risk factor according to our primary analysis. For the same reason, results of routine blood examinations were not requested for individuals in validation cohort 2. The detailed baseline characteristics of patients in the primary and validation cohorts are listed in Table 1 and Supplementary Table 1.

### CRT in the Primary Cohort

In total, 397 cases (12.7%) of CRT were recorded in our study, with an incidence of 1.80 per 1,000 catheter days. The most common CRT site was the subclavian vein, which accounted for 52.14% (207/397) of the CRT cases. Moreover, 33.75% (134/397) of the CRT cases involved multisite thrombosis. Detailed information on the distribution of thrombosis is shown in Table 2.

**Table 2:** Catheter-related thrombi in the primary and validation cohorts.

**Catheter-related thrombi**	**Primary cohort (*n* = 397)**	**%**	**Validation cohort 1 (*n* = 96)**	**%**	**Validation cohort 2 (*n* = 10)**	**%**
**Site**						
Subclavian vein	207	52.14	73	76.04	3	30.00
Internal jugular vein	34	8.56	12	12.50	0	0.00
Axillary vein	146	36.78	14	14.58	3	30.00
Basilic vein	138	34.76	14	14.58	7	70.00
Brachial vein	31	7.81	1	1.04	1	10.00
Other veins of the upper extremity	6	1.51	2	2.08	1	10.00
Femoral vein	6	1.51	0	0.00	0	0.00
Other veins of the lower extremity	8	2.02	0	0.00	0	0.00
Multisite thrombosis	134	33.75	16	16.67	4	40.00

### Development of the CRT Prediction Model

**Figure 1:**
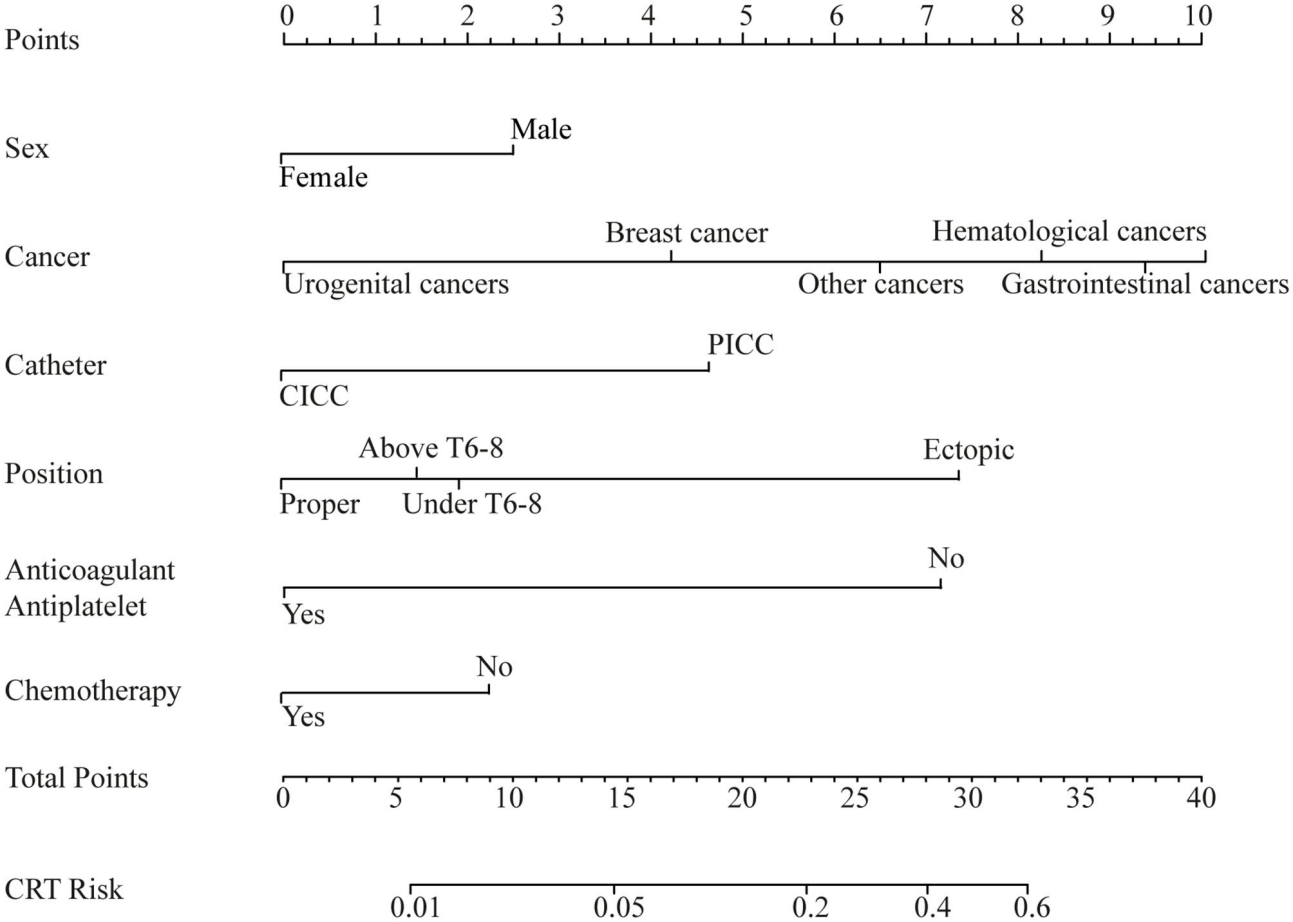
Nomogram. Nomogram of the predicted catheter-related thrombosis (CRT) risk in cancer patients.

The results of the univariate analysis are listed in Supplementary Table 2. The multivariate analysis combined the stepwise forward and backward selection techniques and demonstrated that sex (male vs. female), type of cancer, type of venous catheter used (CICC vs. PICC), position of the catheter tip, chemotherapy (conventional or targeted) initiated at inclusion, and antiplatelet or anticoagulation status at baseline were independent risk factors for CRT (Table 3). These six factors that were verified by the multivariate analysis were included in our new prediction model. The prognostic nomogram that integrated the six independent factors is shown in Figure 1, and the score of the nomogram is shown in Supplementary Table 3.

**Table 3:** Multivariate analysis of the risk of catheter-related thrombosis (CRT).

**Characteristic**	**Non-CRT cases (*****n*** **=** **2,734, %)**		**CRT cases (*****n*** **=** **397, %)**		**Multivariate analysis**	
	***n***	**%**	***n***	**%**	**OR (95% CI)**	***P***
Sex					1.43 (1.06–1.92)	0.018
Male	776	28.38	221	55.67		
Female	1,958	71.62	176	44.33		
Type of cancer						0.000
Breast cancer	1,336	48.87	97	24.43	2.82 (1.50–5.29)	0.001
Thoracic cancer	512	18.73	151	38.04	6.25 (3.35–1.68)	0.000
Gastrointestinal cancers	415	15.18	115	28.97	5.80 (3.08–10.93)	0.000
Urogenital cancer	72	2.63	16	4.03	Ref	Ref
Hematological cancers	39	1.43	6	1.51	5.53 (2.46–12.46)	0.000
Other tumors^#^	360	13.17	12	3.02	3.15 (1.09–9.14)	0.035
Type of venous catheter					2.64 (2.06–3.39)	0.000
CICC	1,505	55.05	116	29.22		
PICC	1,229	44.95	281	70.78		
Position of the catheter tip						0.035
Proper position (T6–8)	2,497	91.33	352	88.66	Ref	Ref
Improper position						
Above T6–8	202	7.39	32	8.06	1.29 (0.85–1.95)	0.231
Under T6–8	24	0.88	5	1.26	1.73 (0.61–4.90)	0.305
Not in the superior vena cava	11	0.40	8	2.02	3.63 (1.35–9.81)	0.011
Chemotherapy (conventional or targeted) initiated at inclusion	2,506	91.66	357	89.92	1.77 (1.21–2.59)	0.003
Antiplatelet or anticoagulation status at baseline	37	1.35	2	0.50	0.25 (0.07–0.84)	0.025

*Ref, reference*.

# *Head and neck cancer, melanoma, sarcoma, neuroendocrine tumor*.

**Figure 2:**
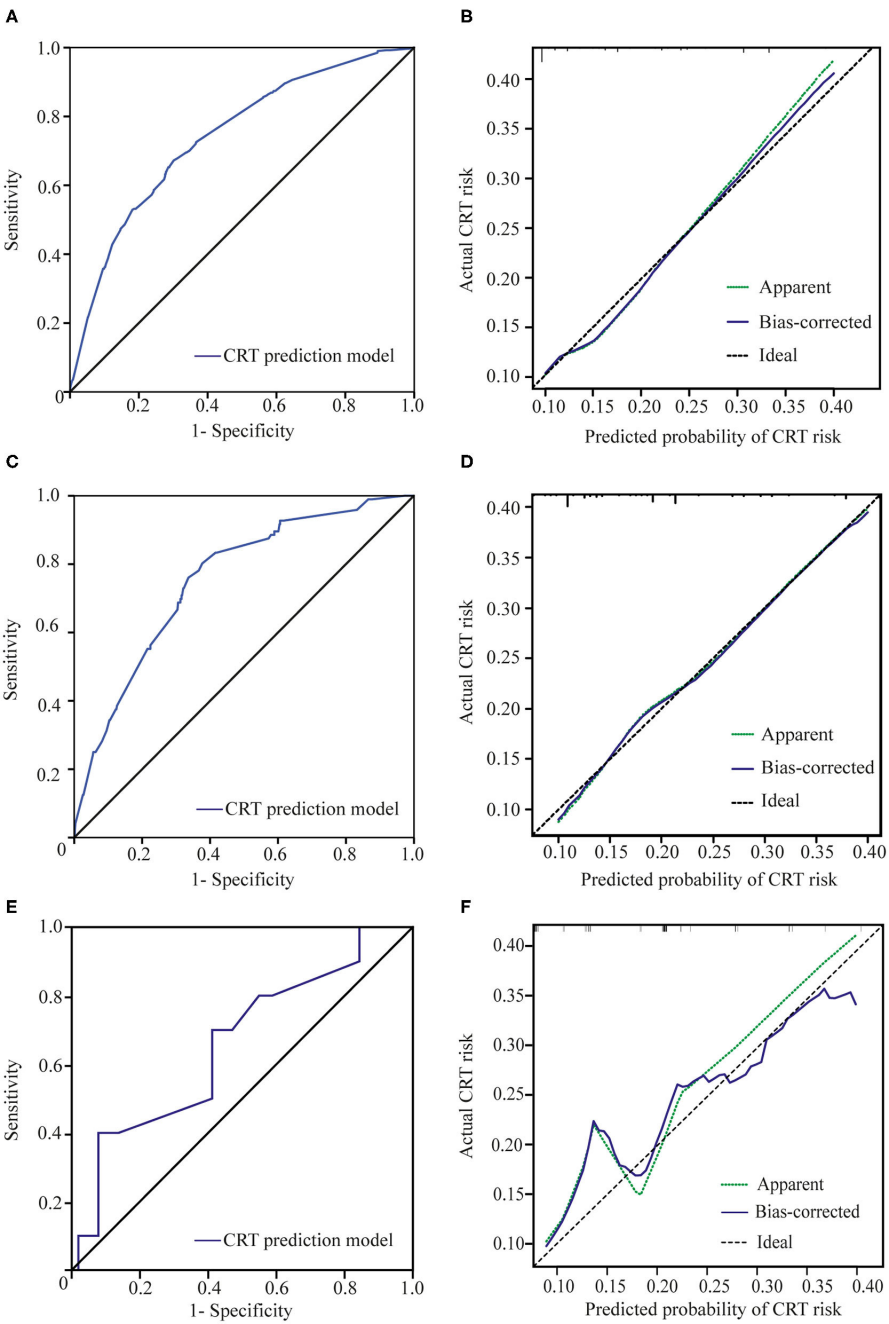
Receiver operating characteristic (ROC) curves and calibration plots. **(A)** ROC curve of the CRT prediction model in the primary cohort. **(B)** The calibration plot for the risk of CRT in the primary cohort showed optimal agreement between the prediction and actual observation. The apparent line is the in-sample calibration. The bias-corrected line is derived via 1,000 repetitions of bootstrapping. The ideal line represents a perfect prediction (the predicted probability equals the observed probability). **(C)** ROC curve of our prediction model in validation cohort 1. **(D)** The calibration plot in validation cohort 1 also showed optimal agreement between the prediction and actual observation. The apparent line is the in-sample calibration. The bias-corrected line is derived *via* 100 repetitions of bootstrapping. The ideal line represents a perfect prediction (the predicted probability equals the observed probability). **(E)** ROC curve of the CRT prediction model in validation cohort 2. **(F)** The calibration plot for the risk of CRT in validation cohort 2 showed good agreement between the prediction and actual observation. The apparent line is the in-sample calibration. The bias-corrected line is derived *via* 40 repetitions of bootstrapping. The ideal line represents a perfect prediction (the predicted probability equals the observed probability).

The area under the ROC curve of our new prediction model was 0.741 (CI: 0.715–0.766) in the primary cohort (Figure 2A). The coefficient of determination (*R*^2^) of 0.138 and the results of the Pearson goodness-of-fit test and Hosmer–Lemeshow goodness of fit test were not significant (*P* = 0.138, *df* = 6). The calibration plot for CRT risk showed optimal agreement between our model's prediction and the actual observation (Figure 2B).

**Figure 3:**
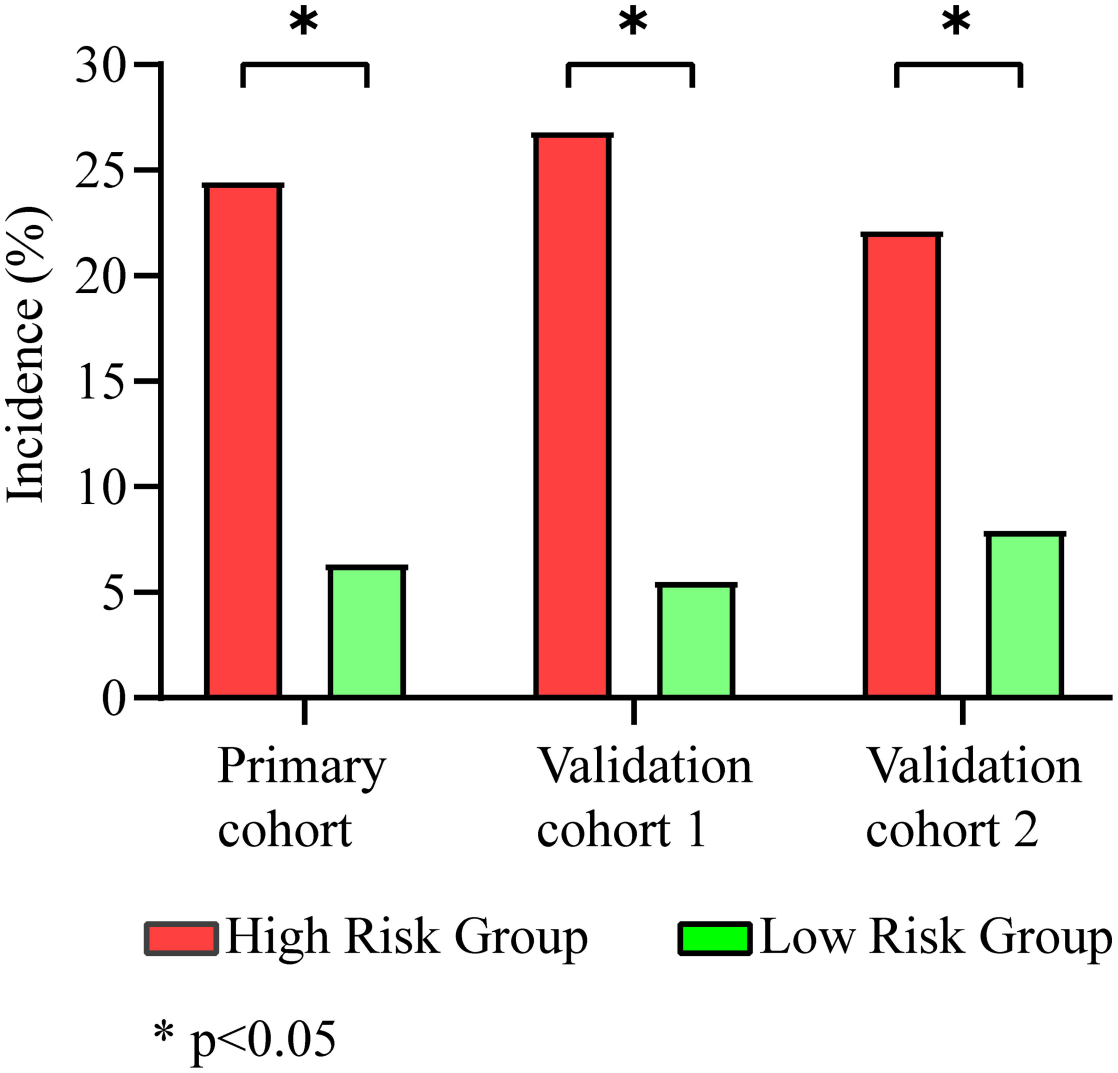
Incidence of CRT in different groups. The high-risk group had a higher incidence of CRTs than the low-risk group in the primary cohort (24.5 vs. 6.4%), validation cohort 1 (26.8 vs. 5.6%), and validation cohort 2 (22.2 vs. 8.0%).

The maximum Youden index was 0.371 (sensitivity, 67.3%; specificity, 69.8%) at a score of 19.6. Patients with a score higher than 19.6 were considered at high risk of CRT. The incidence of CRT in the high-risk group was 24.5% (267/1,092), which was significantly higher than that in the low-risk group (6.4%, 130/2,039) (*p* < 0.001) (Figure 3).

### Validation of the Clinical Prediction Model

Using our new prediction model, we first tested the performance of the model in a prospective validation cohort (validation cohort 1). The ROC curve and calibration plot of validation cohort 1 are shown in Figures 2C,D. The area under the ROC curve was 0.754 (CI: 0.704–0.803), the coefficient of determination (*R*^2^) was 0.214, and the results of the Pearson and Hosmer–Lemeshow goodness-of-fit tests were not significant (*p* = 0.875, *df* = 7). These results indicate that our model shows good discrimination and calibration. The incidence of thrombosis in the low-risk group was 5.6% (23/413), which was significantly lower than that in the high-risk group (26.8%, 73/272) (*p* < 0.001) (Figure 3).

Validation cohort 2 was a smaller cohort, and the area under the ROC curve also reached 0.658 (CI: 0.470–0.845). The calibration curve showed good agreement with the actual CRT risk (Figures 2E,F). The incidences of thrombosis in the low- and high-risk groups were 8.0% (2/25) and 22.2% (8/36), respectively (*p* < 0.001) (Figure 3).

### Comparison of the CRT Prediction Model and the Khorana Risk Score Model

**Figure 4:**
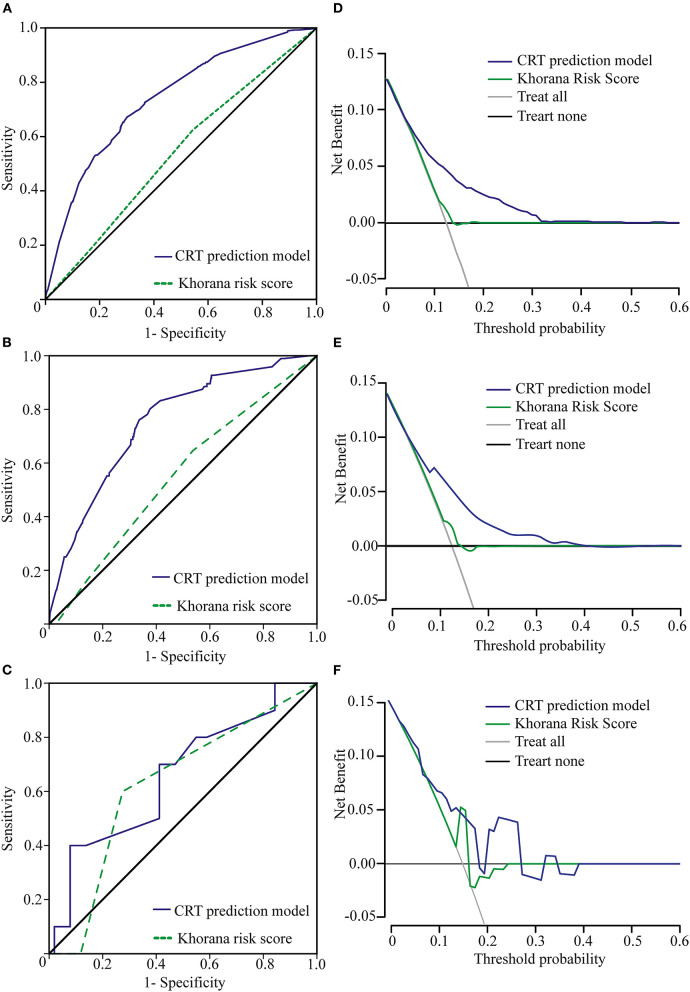
Comparison of ROC curves and net benefits between the Khorana risk score model and the new CRT prediction model. **(A)** ROC curve of the CRT prediction model and the Khorana risk score model in the primary cohort. **(B)** Our new model is the higher line on the decision curve, which indicates that the CRT prediction model leads to a higher net benefit than the Khorana risk score model in the primary cohort. **(C)** ROC curve of the CRT prediction model and the Khorana risk score model in validation cohort 1. **(D)** Our new model is the higher line on the decision curve, which indicates that the CRT prediction model leads to a higher net benefit than the Khorana risk score model in validation cohort 1. **(E)** ROC curve of the CRT prediction model and the Khorana risk score model in validation cohort 2. Due to the small sample size of validation cohort 2, there was no significant difference in the area under the ROC between the two groups. **(F)** Due to the small sample size of cohort 2, our new model is basically the higher line on the decision curve.

The Khorana risk score model is commonly used as a prediction tool for VTE in cancer patients. It is composed of five risk factors: the site of cancer, prechemotherapy platelet count, hemoglobin level (and/or use of erythropoiesis-stimulating agents), prechemotherapy leukocyte count, and body mass index (BMI) (16). The site of cancer (the type of cancer) was the only risk factor included in our analysis and incorporated into our new model for predicting CRT risk. However, in terms of predicting CRT risk in cancer patients, the Khorana risk score model did not perform very well. In our primary cohort, the area under the ROC curve of the Khorana risk score model was 0.539 (CI: 0.509–0.569), and the area under the ROC curve of our CRT prediction model was 0.741 (CI: 0.715–0.766), which was significantly higher than that of the Khorana risk score model (*p* < 0.001) (Figure 4A). The same result was obtained in validation cohort 1; the area under the ROC curve of the Khorana risk score model was 0.551 (CI: 0.490–0.611), and the area under the ROC curve of our CRT prediction model was 0.755 (CI: 0.705–0.804) (*p* < 0.001) (Figure 4B). However, the advantages of our new model over the Khorana risk score model were not statistically significant in validation cohort 2 (Figure 4C).

On the decision curve, our new model was the higher line, indicating that our new prediction model leads to a higher net benefit than the Khorana risk score model for cancer patients with catheters (22). The net benefit represented the balance between CRT risk and the potential cost of unnecessary thromboprophylaxis (Figures 4D–F).

## Discussion

According to the Global Cancer Statistics, 18.1 million new cancer cases and 9.6 million cancer-related deaths occurred globally in 2018, and Asians accounted for nearly 50% of new cancer cases and nearly 70% of cancer-related deaths (23). In line with this global report, 4.3 million new cancer cases were reported in China in 2018 (24). Concerning the treatment of tumors, most patients inevitably need to use a venous catheter, resulting in a very large number of cancer patients at risk of CRT.

Several CRT prediction models have been reported but not externally validated (25, 26), and they did not give special attention to the cancer status/type. A number of prediction models for cancer-associated venous thromboembolism have been proposed in recent years (16, 27, 28). However, the catheter-related risk factors were not taken into consideration when developing these models. A CRT risk analysis in breast cancer patients and a CRT predictor analysis in cancer patients with central venous ports have also been reported in the literature (29, 30). However, without a scoring system to stratify individuals into different risk groups, knowing only the risk factors will make it difficult to put the knowledge into practice. Moreover, limited to a specific type of cancer or a certain type of catheter may increase the accuracy of a prediction model for a certain group of patients, but at the same time, it could be a significant trade-off of the scope of applications.

The duration of the present study was 4.5 years. In total, 3,877 patients were enrolled, and 36 variables of cancer patients were analyzed. All patients underwent vascular ultrasound to avoid false negatives, and all data were carefully documented. We developed a new clinical prediction model for CRT in cancer patients. Our novel prediction model was externally validated in two independent cohorts. Above all, we consider our risk prediction model to be reliable, and this approach is worth popularizing in clinical practice.

Potentially serious and life-threatening complications of CRT could lead to an inability to obtain blood samples, delays in therapy, prolonged hospitalization, frequent catheter replacement, and even death (31). Catheter-associated thrombosis is one of the most important complications. The incidence of CRT in cancer patients from different series varies from 2.4 to 61.5%, and the incidence of symptomatic thrombi also varies widely, from 0.3 to 28% (4, 5). Our study revealed that the total incidence of CRT was 12.7% (1.80/1,000 catheter days), consistent with previous research.

The risk factors for CRT reported in previous studies were numerous and controversial. A meta-analysis noted that none of the 25 studies included the same high-risk factors for CRT (32), showing how difficult it is to establish a CRT prediction model. Some scholars classified CRT risk factors as follows: patient, biomarker, treatment, catheter, technical, and vessel related (33). We found in our research that sex, the type of cancer, the type of venous catheter used, the CRT position of the catheter tip, chemotherapy initiated at inclusion, and the antiplatelet or anticoagulation status at baseline are closely related to CRT.

Regarding the incidence of CRT in patients with different types of cancer, we found that patients with thoracic cancer, gastrointestinal cancers, and hematological cancers were at a relatively high risk of thrombosis, while the lowest incidence was observed in patients with urogenital cancer. Lung cancer, gastrointestinal cancer, and lymphoma were associated with a high risk of CRT, which was in agreement with previous findings. However, the risk of CRT in patients with urogenital cancer was not consistent with that reported in the literature (16), which may be related to the inconsistency in the enrollment criteria and the methods of thrombus detection used.

The advantages and disadvantages of different catheters have been discussed for a long time. At present, the merits and demerits of PICCs and CICCs are still controversial. Owing to the limited evidence available, there is no clear preference between PICCs and CICCs for treatment in clinical guidelines (4, 5). In most studies, the use of PICCs was associated with higher rates of thrombosis (34–36). However, Cai et al. reported the opposite conclusion (37). Consistent with most previous studies, our multivariate analysis showed that patients with PICCs are more than twice as likely to develop thrombosis than patients with CICCs. Implanted access ports (PORTs) have been another commonly used device in recent years. The safety of the infusion port has been widely recognized. Compared with PORTs, PICCs are associated with a higher risk for catheter-related DVT and other adverse events (38, 39).

The relationship between the exact tip position of the catheter and thrombosis has rarely been reported. The tip of the catheter should be located at the lower third of the superior vena cava, at the cavoatrial junction, or the upper third of the right atrium. Improper positioning of the catheter tip is associated with a high risk of malfunction, venous thrombosis, vessel erosion, visceral complications, and other complications (34, 40, 41). Our study found that abnormal catheter positioning was closely related to thrombosis. Not locating the catheter tip in the superior vena cava posed three times greater CRT risk than locating the tip in a proper location. Currently, postoperative standard chest X-ray is an economical and reliable way to determine the location of the catheter (4, 5). However, even if the initial position is correct, mispositioning may occur later for many reasons, such as obesity, body movements, breathing movements, or variations in the venous anatomy (congenital or acquired) (42).

We found that chemotherapy was a risk factor for CRT, which is consistent with a few previous studies (43, 44). Some articles have shown that the CRT incidence in patients with parenteral nutrition is significantly high (45), but a similar conclusion was not reached in our study. No associations between radiotherapy, anti-infective therapy, and CRT were observed.

Our study also found that short catheter days may be protective for CRT. However, this finding could be biased. Because the catheter was removed ahead of time if a thrombus was detected, this reduced catheter days. Thus, we did not include catheter days in the multivariate analyses.

Some high-risk factors that were reported in previous studies were not identified in this study. For example, BMI, platelet count, and hemoglobin, which were included in the Khorana risk score as risk factors for VTE (16), were not associated with CRT risk in our model. A few recognized high-risk factors for chronic cardiovascular diseases, such as smoking, hypertension, and diabetes, have not been identified as high-risk factors for CRT (46). One possible explanation is that, unlike cardiovascular diseases, which are caused mainly by long-term chronic conditions, for patients who have a relatively short catheterization duration, their risk is closely related to the primary tumor and characteristics of the catheter itself. Surprisingly, the D-dimer concentration had no effect on predicting CRT. Although many studies have agreed that the D-dimer concentration is positively correlated with symptomatic DVT, the performance of the CRT prediction model based on the D-dimer concentration was poor in cancer patients (47). The main reason for this finding was that the D-dimer concentration is generally high in cancer patients, especially in patients with terminal-stage cancer.

As mentioned above, the Khorana risk score is widely used to predict the risk of VTE in cancer patients. As shown in our study, the use of the Khorana risk score in assessing CRT risk is extremely limited. Our risk prediction model showed good discrimination and calibration. Cancer patients were accurately divided into a high- or low-risk group. External validation also confirmed the reliability of our model.

Current guidelines do not recommend routine prophylaxis with anticoagulants to prevent CRT. The approach of systemic anticoagulation has not shown any solid evidence of decreasing CRT incidence (4, 5). Our next research goal may be to evaluate the benefits of routine prophylaxis in high-risk patients selected by our model.

This study has several limitations undermining its generalizability. First, the primary cohort in this open-label study was from a single center. It is possible that our study does not reflect the full spectrum of cancer patients. Second, this study was observational, and whether early interventions, such as antiplatelet therapy, would have changed the clinical outcomes is still unknown. Further investigations should be carried out.

## Conclusions

In conclusion, we developed and validated a new risk prediction model for CRT in cancer patients. It is easy to use and available as a paper-based nomogram. This simple model was able to accurately discriminate patients at high and low risks of CRT. The use of this model could help clinicians make decisions regarding prophylaxis in cancer patients and provide clues for the early monitoring and detection of thrombotic events.

“*A stitch in time may save nine.”—Thomas Fuller's Gnomologia, Adagies and Proverbs, Wise Sentences and Witty Sayings, Ancient and Modern, Foreign and British, 1732*.

## Data Availability Statement

All datasets presented in this study are included in the article/Supplementary Material.

## Ethics Statement

The studies involving human participants were reviewed and approved by Institutional Review Boards of Cancer Hospital, Chinese Academy of Medical Sciences. Written informed consent for participation was not required for this study in accordance with the national legislation and the institutional requirements. Written informed consent was not obtained from the individual(s) for the publication of any potentially identifiable images or data included in this article.

## Author Contributions

BL, FM, and YZ conceptualized and designed the study. JX and ZYu were responsible for the catheterization. JX, YZ, BL, XS, XL, and YW acquired the data. BL, ZH, and ZYi analyzed the data. JX and ZYi gave administrative, technical, or material support. BL drafted the manuscript. All authors were responsible for the critical revision of the manuscript and reviewing.

## Conflict of Interest

The authors declare that the research was conducted in the absence of any commercial or financial relationships that could be construed as a potential conflict of interest.

## Supplementary Material

Supplementary Figure 1 Flow chart. Flow chart of patient enrollment.

Supplemental Table 1 Baseline examination of cases.
