# Supplementary material for: Development and Validation of a New Clinical Prediction Model of Catheter-Related Thrombosis Based on Vascular Ultrasound Diagnosis in Cancer Patients
Source: Front Cardiovasc Med. 2020 Oct 26;7:571227. doi: 10.3389/fcvm.2020.571227 (PMC7649194; doi:10.3389/fcvm.2020.571227)
Supplement: Supplemental Table 1 — Baseline examination of cases. [file Table_1.docx]

Supplemental Table 1. Baseline examination of Cases

| No. | Characteristic | Primary Cohort (n=3131) | % | Validation Cohort 1 (n=685) | % | Validation Cohort 2 (n=61) | % |
| --- | --- | --- | --- | --- | --- | --- | --- |
| Baseline examination | |  |  |  |  |  |  |
| 31 | White blood cell count (×10^9^/L, Mean ± SD) | 6.02±4.94 |  | 6.01±2.10 |  | 6.27±2.04 |  |
| 32 | Neutrophil count (×10^9^/L, Mean ± SD) | 4.25±5.02 |  | 3.88±1.86* |  | 3.90±1.52 |  |
| 33 | Hemoglobin count (g/L, Mean ± SD) | 112.6±15.7 |  | 123.74±17.44* |  | 132.0±16.9* |  |
| 34 | Platelet count (×10^9^/L, Mean ± SD) | 224.0±90.3 |  | 246.76±85.97* |  | 251.1±70.4* |  |
| 35 | Lymphocyte count (×10^9^/L, Mean ± SD) | 1.25±0.80 |  | 1.57±0.87* |  | 1.69±0.75* |  |
| 36 | D-Dimer (mg/L) | 1.14±2.76 |  | NA |  | NA |  |
|  |  |  |  |  |  |  |  |

NA: not available. #: Head and Neck Cancers, Melanoma, Sarcoma, Neuroendocrine tumor. * Difference was observed between the Primary cohort and Validation cohort, p<0.05.

Supplemental Table 2. Univariate analysis of the risk of CRT

| Characteristics | Univariate analysis | |
| --- | --- | --- |
|  | OR (95% CI) | P |
| Age (<60 vs ≥60) | 1.64(1.32-2.03) | **0.000** |
| Sex (Male vs Female) | 3.17(2.56-3.93) | **0.000** |
| Type of cancer: |  |  |
| Breast cancer | 2.18(1.18-4.01) | **0.013** |
| Thoracic cancer | 8.85(4.84-16.17) | **0.000** |
| Gastrointestinal cancers | 8.31(4.51-15.32) | **0.000** |
| Urogenital cancer | Ref | **Ref** |
| Hematological cancer | 6.67(3.03-14.69) | **0.000** |
| Other tumors^#^ | 4.62(1.64-12.98) | **0.004** |
| Stage (Localized vs Advanced) | 1.93(1.55-2.41) | **0.000** |
| KPS (>80 points vs ≤80 points) | 1.63(1.28-2.07) | **0.000** |
| Comorbidity |  |  |
| Hypertension | 1.13(0.88-1.43) | 0.336 |
| Diabetes mellitus | 1.17(0.85-1.62) | 0.332 |
| Coronary heart disease | 0.88(0.49-1.59) | 0.677 |
| Cerebral infarction | 0.61(0.24-1.53) | 0.293 |
| Deep venous thrombosis | 0.49(0.06-3.74) | 0.492 |
| Arrhythmia | 1.04(0.55-1.98) | 0.908 |
| Smoking history | 2.77(2.23-3.44) | **0.000** |
| Drinking history | 2.66(2.13-3.32) | **0.000** |
| BMI (＜25 vs ≥25 ) | 0.81(0.65-1.01) | **0.055** |
| Types of venous catheters (CICCvs PICC) | 2.97(2.36-3.73) | **0.000** |
| Insertion side | 0.69(0.55-0.86) | **0.001** |
| Position of the catheter tip |  |  |
| Proper position (T6-8) | Ref | Ref |
| Improper position |  |  |
| Above T6-8 | 1.12(0.76-1.66) | 0.557 |
| Under T6-8 | 1.48(0.56-3.90) | 0.430 |
| Not in superior vena cava | 5.16(2.06-12.91) | **0.000** |
| Secondary adjustment of catheter position | 1.81(1.02-3.23) | **0.044** |
| Catheterization history | 0.65(0.48-0.89) | **0.007** |
| White blood cell count (×10^9^/L) | 0.99(0.97-1.02) | 0.487 |
| Neutrophil count (×10^9^/L) | 1.00(0.98-1.02) | 0.974 |
| Hemoglobin count (g/L) | 1.00(1.00-1.01) | 0.310 |
| Platelet count (×10^9^/L) | 1.00(1.00-1.00) | **0.220** |
| D-Dimer (mg/L) | 1.03(1.00-1.06) | **0.044** |
| Treatments initiated at inclusion |  |  |
| Chemotherapy (conventional or targeted) | 0.81(0.57-1.16) | **0.249** |
| Radiotherapy | 1.55(1.17-2.05) | **0.002** |
| Parenteral nutrition | 0.55(0.28-1.09) | **0.087** |
| Anti-infective therapy | 1.52(1.13-2.04) | **0.006** |
| others | 0.25(0.06-1.02) | **0.052** |
| Antiplatelet or anticoagulation status at baseline | 0.40(0.12-1.29) | **0.125** |

Ref: set as reference. #: Head and Neck Cancers, Melanoma, Sarcoma, Neuroendocrine tumor.

Supplemental Table 3. Risk factors involved in the prediction model

| Variable | Regression coefficient | Nomogram scores |
| --- | --- | --- |
| Sex | 0.455 |  |
| Male |  | 2.45 |
| Female |  | 0.00 |
| Type of cancer: |  |  |
| Breast cancer | 0.782 | 4.20 |
| Thoracic cancer | 1.860 | 10.00 |
| Gastrointestinal cancers | 1.739 | 9.35 |
| Urogenital cancer | 0.000 | 0.00 |
| Hematological cancer | 1.529 | 8.22 |
| Other tumors^#^ | 1.203 | 6.47 |
| Types of venous catheters | 0.854 |  |
| CICC |  | 0.00 |
| PICC |  | 4.59 |
| Position of the catheter tip |  | 0.00 |
| Proper position (T6-8) | 0.000 | 0.00 |
| Improper position |  |  |
| Above T6-8 | 0.247 | 1.33 |
| Under T6-8 | 0.362 | 1.95 |
| Not in superior vena cava | 1.371 | 7.37 |
| Chemotherapy (conventional or targeted) initiated at inclusion | 0.412 |  |
| Yes |  | 2.22 |
| No |  | 0.00 |
| Antiplatelet or anticoagulation status at baseline | -1.336 |  |
| Yes |  | 0.00 |
| No |  | 7.18 |

#: Head and Neck Cancers, Melanoma, Sarcoma, Neuroendocrine tumor.
